# Identity Work: A Qualitative Study of Residents’ Experiences Navigating Identity Struggles

**DOI:** 10.5334/pme.1549

**Published:** 2024-11-11

**Authors:** Adam P. Sawatsky, Caroline L. Matchett, Frederic W. Hafferty, Sayra Cristancho, William E. Bynum, Jonathan S. Ilgen, Lara Varpio

**Affiliations:** 1 Division of General Internal Medicine, Mayo Clinic, Rochester, MN, USA; 2 Division of Gastroenterology and Hepatology, Mayo Clinic, Rochester, MN, USA; 3 Program on Professionalism and the Future of Medicine, Accreditation Council for Graduate Medical Education, USA; 4 Department of Surgery and Faculty of Education and scientist, Centre for Education Research & Innovation, Schulich School of Medicine and Dentistry, Western University, London, ON, Canada; 5 Department of Family Medicine and Community Health, Duke University School of Medicine, Durham, NC, USA; 6 Department of Emergency Medicine, University of Washington School of Medicine, Seattle, Washington, USA; 7 Division of Emergency Medicine, Children’s Hospital of Philadelphia, Philadelphia, PA, USA

## Abstract

**Introduction:**

Medical training traditionally holds a deterministic view of professional socialization wherein many medical learners struggle to construct a professional identity. Previous research has demonstrated the dysfunctional norms and conflicting ideologies that create identity struggle, disproportionally affecting women and individuals underrepresented in medicine. Symbolic interactionism can help explain identity struggles, emphasizing the influence of socio-contextual factors on identity construction. The purpose of this study was to explore how residents navigate identity struggles during residency training.

**Method:**

We conducted a qualitative exploration of 12 residents in three specialties at three academic institutions in the United States. Participants engaged in rich picture drawings followed by one-on-one interviews. We coded transcript data and met regularly to identify themes related to residents’ experiences with navigating professional identity struggles.

**Results:**

We identified three main themes on navigating identity struggles: the weight of identity work, the isolating nature of identity work, and the navigation that occurs with and against socio-contextual currents. Residents described identity work as navigation like a boat at sea. This work felt weighty and at times overwhelming and residents often felt unable to discuss their identity struggles with others. Residents utilized what agency they had to either navigate with the current, navigating towards acceptable—albeit imperfect—paths forward, or attempting to go against the current to forge new paths through resistance.

**Discussion:**

This study highlights how context enables and constrains identity construction, how contextual constraints can create dissonance between identities, and the considerable effort required to reconcile dissonance and construct professional identities. Training program adjustments, enhanced resident support, and cultural shifts are required to sustain residents’ identity work. Medical professionals should engage in collective identity work to reimagine the profession’s identity by addressing dysfunctional cultural norms.

## Introduction

Professional identity formation has been suggested as a framework for thinking about the holistic development of learners into medical professionals [1,2]. Cruess et al. have proposed a definition of professional identity—“a representation of self, achieved in stages over time during which the characteristics, values, and norms of the medical profession are internalized, resulting in an individual thinking, acting, and feeling like a physician [3, p. 1447].” This definition, adapted from sociologist Robert Merton [4], reflects a deterministic view of socialization—one where social institutions (e.g., education) reproduce social values and norms that shape how individuals integrate into society [5]. When applied to professional identity formation, this perspective frames medical education as reproducing the medical profession by ingraining specific values and norms in learners who will, in turn, reinforce them during future medical practice. This orientation on socialization and professional identity formation, however, lacks critical attention to issues of power, individual agency, and conflict within the socialization process [5]. It also views values and norms as static and unchanging [5]. Studies examining socialization and identity formation using other perspectives have highlighted the challenges learners face to construct a professional identity during medical training [6,7,8,9,10,11,12,13]. While socialization into the medical profession includes the internalization of many positive values and norms, it also perpetuates a culture with dysfunctional norms—including perfectionism, intolerance of uncertainty, and strict hierarchies [6]—and conflicting ideologies [7]. These dysfunctional norms and conflicting ideologies can make it difficult for learners to construct professional identities that align with their unique subjectivity [7]. Many learners describe losing their sense of self during medical training [8], a phenomenon that disproportionally affects women and individuals underrepresented in medicine [9,10,11,12]. Increasingly, physicians find their work environments threatening to their identities [13], contributing to lower job satisfaction and higher burnout [14,15]. It seems, therefore, that socialization into organizations with dysfunctional norms and practices is a factor contributing to physician burnout [16].

In contrast to deterministic views of socialization, symbolic interactionism highlights how professional identity formation can be a dynamic, give-and-take construction process [17]. Becker et al. used symbolic interactionism to demonstrate how medical students drew on social interactions to make decisions that reflected the intersection between *their personal* values and aspirations and *the profession’s* norms and values [18]. Haas and Shaffir extended this work, demonstrating how medical students worked to manage the impressions of others to gain legitimacy as professionals [19]. More recently, outside of medicine, Costello explored professional socialization and demonstrated that the work to navigate dissonance between personal and professional identities came at the cost of academic success and led to feelings of disappointment, depression, and anxiety [20]. Therefore, from a symbolic interactionist perspective, identities are constructed by individuals who harness their agency and creativity, and who are also influenced by social norms, values, expectations [21]. Identities flow from and are constructed through social interactions; an individual can have multiple identities associated with the many social commitments and roles they inhabit within society (e.g., medical learner, parent, nonbinary partner, Hispanic, Muslim, etc.) [22]. These roles and commitments—and the identities to which they are linked—influence individuals’ social behavior and whether they act in accordance (or not) with the expectations of those social roles and commitments [22]. For example, in a clinical encounter, the resident may choose to foreground their Hispanic identity to build rapport with a patient with a similar identity.

Building on symbolic interactionism, the identity work perspective describes the activities individuals undertake to create, present, and sustain identities [23,24]. Identity work is shaped by socio-contextual factors and intensifies when there are tensions within and between identities [23,24]. For example, large scale changes in the organization of medical work can force individual physicians to reconstruct their professional identities to align with these systemic changes (e.g., the increasing complexity of healthcare delivery has led to a shift in professional identity as a “systems citizen” [25]) [26,27]. Even normal transitions, like entering residency, necessitate identity work as trainees customize their general physician identity to accommodate a new specialist identity [28]. Furthermore, studies using critical theory have revealed that minoritized physicians’ identity work entails facing systemic injustices and stereotype threats, as well as the additional work of racial uplift and mentoring other minoritized physicians [12,29]. Importantly, while everyone engages in identity work, it intensifies during times of change in or strain on identities. Using a symbolic interactionist perspective on identity thus allows us to explore the experience of identity struggles at the level of the individual, at the level of their social interactions, and within the social contexts that both shape and are shaped by their interactions.

In previous work, we examined social forces that shaped residents’ identity construction [7]. Residency in the United States^1^ is a critical time in the construction of professional identities because residents are exposed to multiple tensions within and between the ideals and the realities of medical practice [30,31]. Our previous research demonstrated how the ideology of medicine—the system of norms, ideas, beliefs, and attitudes that drive medical practice—shapes medical learners as they internalize this ideology while constructing their professional identities [7,32]. Adopting this ideology can generate significant identity struggles as learners grapple with the demands of the work and perfectionism, the subjugation of personal identities to their developing professional identity, and the constraints inherent in the reality of medical practice that limit their ability to live their values [7]. While this research elaborated the nature of residents’ identity struggles, it did not examine how they navigated these identity struggles. In this study we examined how residents navigate moments of identity struggle in their residency training experiences.

## Methods

We conducted a qualitative exploration of residents’ experiences with identity struggles in three different specialties at three academic institutions in the United States. We used reflexive thematic analysis [33] and acknowledged that our research was informed by the symbolic interactionist perspective on identity and socialization [20,21,22,34,35]. Within the symbolic interactionist tradition there are many avenues for exploring identity issues; we chose a constructivist stance to the exploration of identity struggles, focusing more on the individuals’ interpretations of their social context than the social processes (i.e., performances, uses of language) that are used to construct identities. To clarify our use of concepts, we have provided a glossary of terms (see Table 1).

**Table 1:** Table Glossary of Terms.


**Identity**	Represents an individual’s response to the question: “Who are you?” It can be individual, relational, or related to larger social categories (e.g., like a profession). It includes views of self as well as how one acts in individual interactions and within social groups. Identities are infused with both personal and social meanings [36].

**Profession**	A profession is defined by: A body of knowledge and skills which is officially recognized as one based on abstract concepts and theories and requiring the exercise of considerable discretion. An occupationally controlled division of labor, labor market, credentialing, and associated schooling. An ideology serving some transcendent value and asserting greater devotion to doing good work then to economic reward [37].

**Professional Identity Formation**	Broadly used to describe the topic of research within the health professions education literature that refers to the process of development of a “representation of self, achieved in stages over time during which the characteristics, values, and norms of the medical profession are internalized, resulting in an individual thinking, acting, and feeling like a physician [3].” This work is often supported by a broad range of theories that offer diverse perspectives on the content of identities and the processes though which they emerge [38].

**Identity Struggle**	The process of working through challenges in the construction of one’s personal and professional identities [7].

**Symbolic Interactionism**	A sociological theory focused on micro-level social interactions with three main premises: Human beings act towards things on the basis of the meanings that things have for them. The meaning of such things is derived from, or arises out of, the social interaction that one has with one’s fellows. These meanings are handled in, and modified through, an interpretive process used by the person in dealing with the things one encounters [34].

**Identity Construction**	From a symbolic interactionist perspective, people construct selves, social worlds, and societies through interaction [21]. Therefore, identities live through social interaction and can be studied through the ways we present ourselves through language and social behavior [38].

**Identity Theory**	Drawing from symbolic interactionism, the main presmise of this theory is that social commitments (i.e., interactional and affective ties to others in social networks) affect the expression of identities, which in turn influences role choice behavior (i.e., opting to meet the expectations of one role rather than another [22].

**Identity Work**	The range of activities individuals engage in to create, present, and sustain personal identities that are congruent with and supportive of the self-concept [39]. This theoretical perspective is based on five sets of assumptions: Selves are reflexive and identities actively worked on, both in soliloquy and social interaction. Identities are multiple, fluid and rarely fully coherent. Identities are constructed within relations of power. Identities are not helpfully described as either positive or authentic. Identities are both interesting per se and integral to processes of organizing [23].


This work was framed by our own experiences as trainees, clinician educators, and health professions education researchers. We engaged in personal and methodological reflexivity throughout the study, providing insights that shaped the research process and findings from the positions of our identities, experiences, and motivations. Three team members (AS, JI, WB) are cisgender, white, male physicians who teach at the graduate medical education level. One team member (CM) is a cisgender, white, female trainee who commented on data from her position as a resident-in-training. Two team members (SC, LV) are cisgender, female Ph.D.-trained researchers with extensive research experience in medical education and qualitative research methods. FH is a Ph.D. medical sociologist who has spent his career applying sociological perspectives to understand medical education and medical professionalism.

We report our findings in accordance with the Standards for Reporting Qualitative Research [36].

### Setting and participants

We used maximum variation sampling [37] to capture and describe professional identity struggles across a wide variety of resident experiences and therefore sought participants from diverse specialties and geographical locations. Residents were invited to participate through a recruitment email. We enrolled residents from internal medicine (n = 7), emergency medicine (n = 3), and family medicine (n = 2) from the following institutions: Mayo Clinic (n = 7), University of Washington (n = 3), and Duke University (n = 2). Ten female and two male residents participated in the study. Participants were diverse in personal characteristics, including race, ethnicity, and religion. Given the personal and identifiable nature of the study data, we do not provide specific demographic information to protect participant anonymity.

### Data collection

Data was collected between July 2021 and June 2022. To solicit insights from participants on identity struggle, we collected data using individual interviews consisting of two activities: drawing a rich picture (up to 30 min) and a one-on-one interview (up to 60 min) with a member of the research team (FH). Rich pictures augment traditional qualitative interviews by helping participants construct and interpret complex experiences and allowing the interviewer to probe deeply about those experiences [38,39,40,41]. We used the rich picture as a visual elicitation technique to help participants give voice to experiences; the products of those techniques (e.g., the rich picture) are not required to be analyzed as a data element [38,42].

We defined identity struggle for participants as “the process of working through challenges in the development of your personal and professional identity, which can lead to progress as you work through and overcome those challenges, or regression as those challenges can lead to an identity crisis.” The interview began with the interviewer (FH) asking the participant to “draw a pictorial representation of a particularly challenging experience during your residency training that represents a specific identity struggle that you have faced.” Thereafter, the interviewer asked participants to narrate elements of the rich picture as a starting point for a semi-structured interview. Interview questions were informed by our previous work on identity formation [31,43], symbolic interactionist perspectives on identity construction and socialization [20,21,22], and the past experiences of members of the research team. The interview protocol explored the ways residents navigated through identity struggles, including the contextual factors that contributed to—or helped resolve—these struggles (see Appendix A).

We concurrently collected and analyzed data, allowing for iterative refinement of the interview guide. We were attentive to instances that challenged the developing insights from our participants’ narratives. To respond to these challenges and deepen the trustworthiness of our data analysis, we sought disconfirming cases—i.e., residents who did not have external signs of identity struggles [44]. To achieve this end, two residents identified by the study team were directly invited to participate in the study.

### Data processing and analysis

Interviews were audio-recorded, transcribed verbatim, and uploaded to a qualitative research software program (Nivo, QSR International Pty Ltd, Burlington, Massachusetts). While the rich pictures were available to all researchers as context for analysis, the interview transcripts were the focus of our analytical attention. Transcripts were initially reviewed and analyzed by the entire research team through open coding, axial coding, analytic memos, and regular team discussions [33].

For this study, we focused our analytical attention on residents’ descriptions of navigating identity struggles. To build on our previous work [7], we reviewed each transcript and created detailed maps outlining each participant’s identity struggles, the context of those struggles, and how they navigated through them. The principal investigator (AS) mapped out each transcript, with each member of the research team mapping out one or more interviews in duplicate. Through group discussions, we explored the coded data and relationships between codes to identify themes related to navigating identity struggles. Throughout this process, the primary investigator created memos to capture analytic insights and relationships among themes. This process harnessed our symbolic interactionist perspectives by highlighting how participants’ individual experiences and interactions represented different aspects of their identity struggles. Through this analysis process, we determined that we had sufficient data to provide conceptual depth to our theoretical understanding of participants’ navigation of identity struggles [45,46].

## Results

Participants’ descriptions of navigating identity struggles centered around three themes: the weight of identity work, the isolating nature of identity work, and the navigation that occurs with and against socio-contextual currents. First, participants communicated the burden of work required to construct a professional identity, highlighting how these processes occurred above and beyond the day-to-day work of patient care and learning. They illustrated the process of working through identity struggles as navigating the challenges of the socio-contextual environment, *“like a boat at sea trying to steer the right course.” (P9)* Second, while this identity work occurred through social interaction, residents most often described navigating identity struggles as a private individual activity, akin to carrying a weight alone. Third, residents utilized the agency available to them either to navigate *with* the current, moving through personally acceptable—albeit imperfect—paths forward, or *against* the current, forging new paths by resisting existing social norms and expectations.

### The Weight of Identity Work

**Figure 1:**
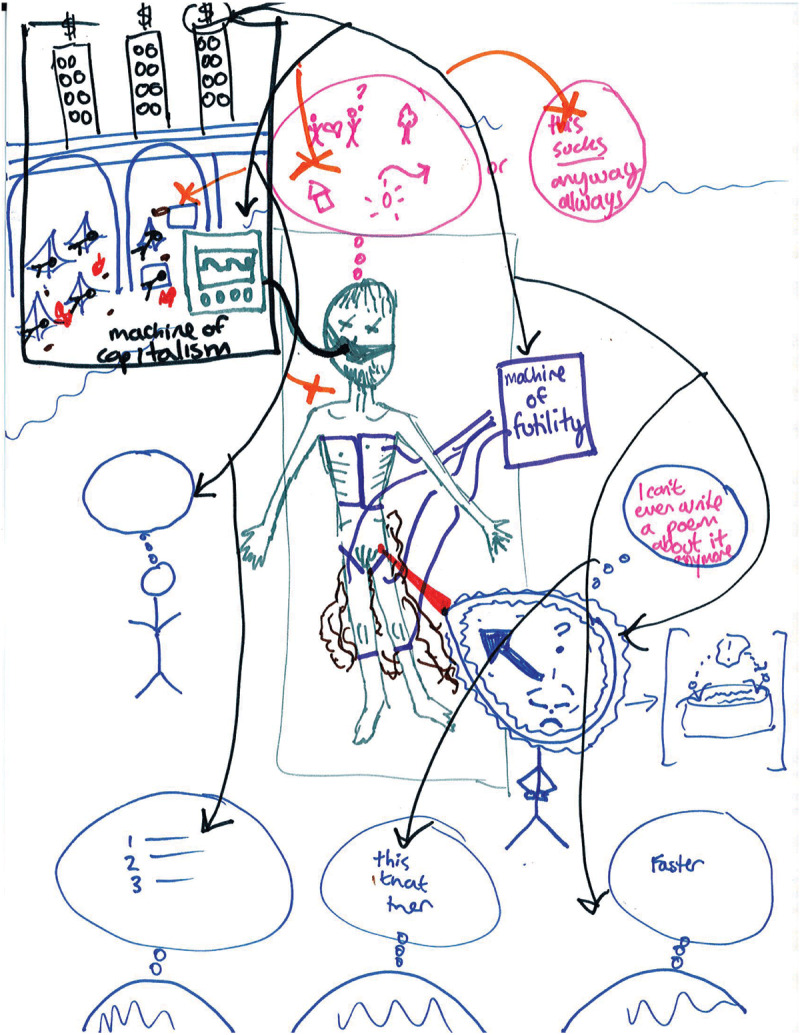
Rich picture example (Interview 5).

No matter how residents framed their identity struggles, navigating them required demanding work. Participants described *“wrestling” (P8)* with the meaning of their professional identity and being ill-prepared for the work required to navigate identity struggles. Whether describing conflict within their professional identity, constraint on their ability to construct their professional identity, or dissonance between their personal and professional identities, participants voiced having to work through tensions to develop their professional identity. As one resident acknowledged: *“there’s a lot of conflict, surprisingly, that comes with this new [physician] identity.” (P11)* Furthermore, the work associated with navigating identity struggles and constructing professional identities occurred above and beyond learning the knowledge and skills of becoming a physician. For example, one resident described being called into a patient’s room to help with resuscitation efforts (see Figure 1). She described feeling confident in the parts of her professional identity associated with her skill at placing central lines: *“One thing I feel good at is procedures.” (P5)* She went on to recall feeling good about herself after successfully placing the central line in the patient; however, stepping back from the procedure, she felt the weight of the resuscitation, the potential futility of some medical interventions, and the likelihood that her successful procedure may not have helped the patient. These reflections sparked internal struggles. She repeatedly described working through those struggles as being “*in a hard spot wrestling with my identity.” (P5)* These irreconcilable considerations were part of the burden of the identity work: *“these issues are weighing heavily on me.” (P8)*

Residents also described the strategic work required to present their professional identities to faculty, colleagues, interprofessional team members, and patients. Wrestling with identity could take the form of navigating the *“social expectations that guide the way we act as physicians…[which] takes its toll.” (P2)* Part of this navigation entailed understanding the social expectations of residents and presenting oneself in socially appropriate ways: *“being a resident is strategic work…yeah, it feels like a game.” (P10)* This impression management work was described as *“the pressure of needing to learn and look good…[it’s] hard to both effectively learn and look good.” (P2)* Therefore, identity work was oriented towards both doing a good job and making positive impressions on others while doing it. In this way, navigating complex and at times conflicting external expectations added to the work of navigating identity struggles.

At times, residents felt overwhelmed by the weight of identity struggles and could feel *“burned out” (P11)* and *“disillusioned” (P10)* when they didn’t measure up to expectations or perform an appropriate professional identity. Residents often interpreted the presence of identity struggles as a personal failure. Sometimes that message was explicitly delivered to the participants by others: *“I was being told that myself wasn’t good enough,” (P2)* but often it was implicit, for example, in a participant who believed *“there is no such thing as good enough” (P10)* in medicine. Adding to that weight was the lack of personal capacity to do identity work. As another participant explained: *“I could barely get a grasp on how to care for someone clinically. I had no bandwidth to think about larger questions and my role in working on those issues, and so, in a way, I gave up on them.” (P8)* Indeed, the overwhelming weight of identity work could prompt a desire to escape the struggle of professional identity navigation. When prompted to elaborate on a door labelled *“escape”* in her rich picture, a resident explained, *“there is a lot of chaos on this side of the door, a lot of constant struggle. Escape felt the most apt word to explain what would happen if I took the door.” (P9)* Taken together, these accounts illustrated a point made by several participants: when the weight of identity work became overwhelming, many residents described thinking about leaving the medical profession.

### The Isolating Nature of Identity Work

Navigating identity struggles often elicited feelings of loneliness for residents. Participants explained that the clinical work environment was not a place to discuss struggles with their personal or professional identities. While participants recalled sharing mundane, *“day-to-day frustrations and struggles” (P7)* with their peers, they rarely talked to each other about the *“overarching…existential” (P7)* issues related to identity work. Instead, they reported unwritten rules about which struggles were appropriate to discuss at work [e.g., *“This patient is really sick, and I am over my head clinically” (P10)*] and which were not appropriate [e.g., *“I am emotionally overwhelmed by these patients” (P10)*]. To mask their struggles, residents often performed as if everything was fine: *“I definitely put on fronts in front of patients, in front of the team, in front of everyone.” (P11)* While residents acknowledged having supportive people outside of work, they felt that those people did not consistently understand the nature of their professional identity struggles. Thus, residents often described being stuck—unable to discuss their identity struggles at work or at home. This led one resident to conceptualize her identity as a *“fractured mosaic” (P12)*, only single pieces of which were seen by other people, compounding her feelings of isolation: *“Everyone gets a bit of the picture…it’s definitely lonely…I’m left to sort it out on my own…figuring it out bit by bit…we’re all left to sort it out on our own” (P12)*. This culture of not talking about identity struggles resulted in residents feeling *“like this must be only happening to me,” (P5)* further entrenching the perception that help was not available, neither inside nor outside of work.

While residents described the isolation of identity struggles, they also discussed breakthrough moments coming from the support of others. For example, when struggling with tensions between her personal and professional identities, one resident appreciated the support from faculty members who gave her a *“glimmer of hope” (P2): “I needed to reclaim myself with the help of others…especially other female physicians. They were more likely to validate the kind of concerns I had for patients*.” *(P2)* These supporters *“gave [her] permission” (P2)* to be herself. In another instance, a participant’s *“senior [resident] took [her] aside” (P11)* to provide support when an attending’s inappropriate behavior was adding to the resident’s identity struggles. Other participants also recognized the support of co-residents. One participant identified a co-resident who had personal and professional identities in common: *“Having this friend has been a treasure for me. She keeps me centered through all the madness…she’s helping me form my identity and stay true to who I am.” (P9)* Although such relationships were difficult to develop and maintain during residency training, they *“made all the difference in the world” (P8)* by reducing the loneliness of engaging in identity work.

### Navigation With and Against Socio-Contextual Currents

The work of constructing a professional identity often entailed navigating unyielding social currents. Residents’ identity work was laden with specific social expectations, career paths, and ways of being a doctor, each constraining their agency to act in line with their desired professional identities. One resident explained that most residents *“get swept into the current and take the path of least resistance. It’s easier to go with the flow than to question things.” (P3)* Not wanting to completely capitulate to unyielding currents, this participant struggled to find the path “*…where I can navigate and figure this out.” (P3)* Residents’ constrained ability to direct their professional identities into desired currents carried several consequences. First, residents expressed having little agency over how they should be or act as a professional. One resident described *“suffocating myself as a person” (P2)* while seeking to fit expectations to *“be a certain way with a certain style and…[to] adhere specifically to that dogma.” (P2)* Second, residents felt they had to navigate through and select among several unsatisfying options. On reflecting on her future professional identity, one resident described that working in an underserved setting *“will help me feel less of an internal, moral dilemma”* (P7), but then lamented that she would only be a *“Band-Aid” (P7)*, offering superficial help instead of her ideal vision of providing comprehensive care. She ultimately decided to work in the hospital where she could *“focus on the medical aspect and fixing problems” (P7)* and ignore the uncomfortable external complexities of social issues and financial cost. Third, residents struggled to forge paths that aligned with their ideal professional identity. One resident abandoned her desired career path because she saw herself “*constantly trying to fight” (P4)* between her professional identity and personal identities. Lastly, some participants explained that the weight of navigating between existing choices was too heavy. One resident felt forced to “*focus on building my clinical skills” (P8)* and resigned that he may never realize his desired professional identity: *“I had many thoughts of, ‘maybe I’m never going to do anything in public health, maybe there isn’t a way to tie these things together.’” (P*8)

Participants explained that wielding their limited agency was akin to resisting the pressures of structurally and culturally embedded currents. This resistance required significant work to *“actively negate the hidden curriculum” (P2)* because *“the [culture of medicine] is hard to push against.” (P8)* One resident described struggling to center her personal faith within of her professional identity: *“I had to fight and advocate…I struggled with that… [my] faith, religion needs to be included.” (P8)* This resistance work could be overwhelming because residents *“don’t have the power to make change” (P4)* and because of the enormity of the struggles: *“You’re facing something so much larger than yourself that you just can’t even begin to address.” (P12)* However, participants found solace in small acts of resistance. Sometimes this resistance was an attempt to maintain the belief that “*you don’t have to go with the grain. The grain clearly will pay more, but the grain is not going to bring you any joy.” (P3)* Other times it was resistance to prescribed ways of practicing medicine: *“I’m not going to read off the script.” (P9)* Resistance could also take the form of residents helping each other maintain their humanity in opposition to medicine’s dehumanizing culture. One participant reflected that *“with wonderful team members, they see that you’re not doing well, they say, ‘Stop. You go eat. I’m taking your pager.’ It’s not that that hidden curriculum isn’t fought at times, but it’s rare.” (P4)* Small acts of resistance, while motivating, had to be weighed against the cost of engaging in them: *“It depends on how I feel that day, whether or not I’m willing to push back…how much emotional battery I have to expend.” (P10)* Whether big or small, acts of resistance enabled residents to leverage agency while navigating with and against the current within identity work, but that resistance took a toll.

## Discussion

Our previous work identified how ideology can create identity struggles in residents as they construct professional identities [7]; this study extends that work by characterizing residents’ experiences with navigating those identity struggles. Through a symbolic interactionist lens, our findings revealed three broad issues relating to identity struggles: context both enables and constrains identity construction, contextual constraints can create dissonance between personal and professional identities, and considerable effort is required to reconcile this dissonance and construct desired professional identities.

First, symbolic interactionism frames professional socialization as a two-way process wherein social contexts provide the building blocks (i.e., enablers) and rules (i.e., constraints) for identity construction. Individuals have agency to accept or resist socializing forces; they can push back against constraints and reshape social norms and values [21]. The identity work perspective, based in symbolic interactionism, recognizes that social constraints are part of all social life and therefore no individual has complete agency over identity construction [23,24]. Social norms both provide the resources for individuals to construct an identity and impose constraints on that construction and expression of identity [23,47]. In this study, residents described their identity work as constrained by pressures to conform and a lack of power, leaving them with little perceived agency to construct professional identities that break existing molds. While our participants described acts of resistance, they recognized the energy cost of this resistance, which other studies show can endanger professional success [20]. However, according to symbolic interactionism, society evolves through individual and collective acts of resistance [21,34]. The capacity for resistance is important for challenging organizational structures and dominant discourses [23,48,49]. While providing adequate space for resistance may be challenging in the regulated and competitive environment of medicine, it is required for residents to effectively engage in identity work. To support professional identity work, training programs should enable residents to harness professional values, norms, and expectations as building blocks, and equip them to push back against the constraints that may be detrimental to identity construction.

Second, our findings demonstrate how contextual constraints create dissonance within and between identities. Early sociological inquiry into the medical profession demonstrated this dissonance as medical students navigated complex training environments and attempted to assimilate professional values and perform as professionals [18,19]. Similarly, narrative analysis of identity construction has revealed tensions between stated professional values and the realities of medical practice [28,50,51]. These studies and others draw our attention to how medical training conveys explicit and implicit messages that emphasize the preeminence of the physician identity [6,7], creating tension as learners subjugate personal to professional identities [7]. Studies using critical theory and acknowledging the intersectionality of identities demonstrate the ways these tensions are compounded, increasing the weight of the work required to navigate identity dissonance [10,11,29]. Indeed, when our findings and those from other studies are considered collectively, tension between identities—and the identity work it requires—appears to be inevitable in medical training.

Lastly, our findings highlight the identity work necessary to navigate socio-contextual constraints and to construct a professional identity. Beyond the demanding work of acquiring a vast array of knowledge and skills, physicians must learn to cope with existential crises [52], the weight of responsibility [53], and the reality of death [54]. In our data, we see that the work associated with constructing a physician identity can feel weighty to residents and can exact a significant physical, emotional, and relational toll. We know that identity work can be more or less arduous, depending on the context; shifting norms and conflicting ideologies can make this identity work particularly challenging [7,47]. However, it is precisely at those moments of struggle that identity work is the most important [24], as it is through difficult experiences that identity transformation can occur [55]. Therefore, struggles will often exist in the journey to become a doctor, and the identity work required to navigate those struggles must be recognized if medical education truly seeks to support professional identity formation.

Critically, individuals require social interaction to navigate identity construction challenges [23,47]. In this study, residents perceived their identity work as isolating, happening at an individual level without consistently available social support. That residents often feel isolated as they develop an identity—an inherently social endeavor—raises questions about our current understanding of and collaborative engagement in learners’ professional identity formation. When examining the existing literature, this incongruence makes sense. Many professional identity formation interventions adopt individual stances toward identity formation, engaging learners in individual reflective writing and narrative reflection [56]. We worry that these efforts alone may worsen the problem, placing the burden of dealing with identity struggles squarely on the learner. Our data and the symbolic interactionist perspective on identity emphasize the need for 1) creating more space in our curriculum for identity work, 2) providing adequate social support for identity work (e.g., with guidance from a coach or mentor [57]), and 3) creating a culture that allows for increased agency in residents’ identity work. We hope this research normalizes identity struggle and helps training programs and educators provide guided support, reflection, and coaching on how to navigate these struggles, setting the stage for ongoing identity construction throughout the career of a physician.

In addition to social support, identity work could be extended to include the collective work required to reimagine medicine’s view of professional identity. The construction of a professional identity is constrained by societal forces, sometimes necessary (e.g., ideals, virtues, competencies, etc.) and sometimes troubling (e.g., ways in which the ideology of medicine calls us into being in impossible ways [7]). Schwalbe and Mason-Schrock extended identity work to encompass the work that a group undertakes to create, maintain, or repair a collective identity—to revise an image of the individuals who belong to the group and provide the resources for individual identity making [47]. Our work, along with the work of others [7,8,9,10,11,12,13,14,15,52,53,54,58] suggests that medicine has a collective identity crisis. The results of this crisis are striking, as a growing number of physicians are reporting an intent to leave practice [59]. Additionally, medical students sense this fracturing identity and increasingly express intentions to work in non-clinical settings after graduation [60]. We must all do the work of reimagining the collective identity of the profession and pushing back on troubling aspects of culture, norms, and ideology that are fracturing physicians’ collective professional identity.

### Limitations

While we believe that insights from this study are transferrable to many contexts, this is not absolute. For instance, our findings may be US-specific given that residency programs are highly variable across national borders. Additionally, as we captured participants’ experiences at a specific time during their training, we were afforded limited glimpses into how these struggles evolved across training experiences. Longitudinal research could offer a more comprehensive view of how residents’ experiences of identity struggles change over time [61]. Lastly, we conducted this study during the COVID-19 pandemic, which may have intensified experiences with identity struggles, impacted the support resources available to participants, or influenced participants’ relationships with others within and outside of medicine.

## Conclusions

Professional identity formation is central to medical education. This study highlights the work that residents must perform to navigate the construction of their professional identities. In performing this identity work, residents struggle because this work is weighty, isolating, and deeply constrained. We urge educators and training programs to programmatically facilitate the navigational work required for learners’ to meaningfully and agentically build a professional identity. In addition, physicians need to do the collective work of creating and sustaining a shared professional identity that supports professional values and continues to serve patients’ needs.

## Additional File

The additional file for this article can be found as follows:

10.5334/pme.1549.s1 Appendix A. Interview Guide.

## Footnotes

In the United States, residents are physicians who have graduated medical school and have continued training within their chosen specialties.
