# Supplementary material for: Identity Work: A Qualitative Study of Residents’ Experiences Navigating Identity Struggles
Source: Perspect Med Educ. 2024 Nov 11;13(1):540–52. doi: 10.5334/pme.1549 (PMC11568810; doi:10.5334/pme.1549)
Supplement: Appendix A. — Interview Guide. [file pme-13-1-1549-s1.pdf]

Introduction:

Welcome and thank you for participating in this interview session. My name is Fred Hafferty, and I will be conducting this interview. The study investigators are interested in exploring some of the challenges you have faced with your developing identity during residency training. This interview session will take place in 2 parts: the first will be to draw a rich picture for up to 30 minutes, based on a prompt, and the second part will be to discuss your picture through a set of interview questions which will last up to 60 minutes.

We want to start by defining some terms related to identity struggle. First, **identity** encompasses the characteristics and personality of you as an individual and your relationships to specific groups of people. Your **professional identity** is your individual identity as a physician, including the different values and roles you take on as a physician and your relationship to other physicians and healthcare workers. Therefore, **professional identity formation** is both an individual development, as you seek to integrate this professional identity with your other identities into a cohesive view of yourself, and a collective development, as you seek to integrate into the community of medicine. We know that this process of professional identity formation presents many joys and challenges, and challenges can be both difficult as well as opportunities for growth. **Identity struggle** is the process of working through those challenges in the development of your personal and professional identity, which can lead to progress as you work though and overcome those challenges, or regression as those challenges can lead to an identity crisis. We are interested in exploring the identity struggles you have faced during your time in residency training.

Rich Pictures

For the first part of this session, we will be asking you to draw a rich picture about your experience with identity struggle as a physician during residency training. A ***rich picture*** is a pictorial representation of a particular situation, including what happened, who was involved, how people felt, how people behaved, and what external pressures were present. We want you to recall and reflect on one experience and use the picture to share your thoughts, feelings, and actions in that experience and how that experience shaped your developing identity. We recognize that drawing may not be something you are comfortable with—we want to reassure you that your artistic ability is not important, but it is the content and reflection that is most important. By drawing your experience, we hope to stimulate reflection in ways that just discussing your experience wouldn't allow.

Interview

Afterwards, you will share your rich picture, and we will use this picture to further explore your experiences. I will be recording the interview for future analysis. The recording will be transcribed, and your information will be de-identified. All of the recordings will be protected by the research team, and all transcriptions will be de-identified for the analysis, in order to protect your anonymity. Your participation and responses will in no way effect your standing within the residency program.

Your participation in this interview is completely voluntary and you do not have to answer any question you do not feel comfortable answering. By remaining on this Zoom meeting, you are giving your consent to participate in this study.

If you have any questions about this research study, you can contact the principal investigator at 6-8373 or sawatsky.adam@mayo.edu. If you have any concerns, complaints, or general questions about research or your rights as a participant, please contact the Mayo Institutional Review Board (IRB) to speak to someone independent of the research team at 507-266-4000 or toll free at 866-273-4681.

Do you have any questions for me about the study before we get started?

Rich Picture Prompt:

Please draw a pictorial representation of a particularly challenging experience during your residency training that represents a specific identity struggle that you have faced. How did that struggle represent a misalignment between who you saw yourself as, and the doctor or person that you aspire to be?

Some possible questions to prompt your reflection: What was the experience? Who was involved? How did you and/or others behave during this experience? What led up to that experience? What things external to yourself contributed to the experience? How did it make you feel? How did this experience change you?

Interview Questions:

A. **Describe the Rich Picture**
    1. Tell me the story behind the drawing.
    2. Describe to me the drawing and explain the elements you included in the picture.
        [Prompt] Make sure to ask about any details that may have been left out of the description
        [Prompt] Make sure to explore relationships between elements within the pictures
        [Prompt] Elaborate on the behaviors or feelings evoked during the situation
        [Prompt] Elaborate on how this experience shaped how you felt about yourself as a doctor
    3. How did this situation affect your view of the doctor you aspired to be? How did this situation affect your view of the person you aspired to be? Where was there misalignment between the person and doctor you were, and the person or doctor you aspired to be?
    4. How did you deal with that misalignment? How did dealing with this challenging situation help to shape the person or doctor that you have become?
    5. What other insights have you gained from reflecting on this situation?

B. Additional Research Questions

From this point on, I want to ask questions to explore more about the idea of "identity struggles" that you have faced during your time in residency training.

1. Who are you striving to become as a doctor? Who are you striving to become as a person? Where do those identities fit together well? Where do those identities not fit together well? Tell me about a time when who you are becoming as a doctor and as a person came into tension with one another. How did it affect you? How did you navigate this tension?
    a. Who do your authentic self to be? How does that that fit (or fail to fit) the professional identity you need to be a doctor?
    b. Where and when do you get to be your authentic self? Why? Why not? How does that change based on the context (e.g. rotation, attending physician, co-residents, etc.)?
2. What have been your biggest challenges that you have faced during your residency training?
    a. What has been the most helpful in facing those challenges?
    b. What has been the biggest obstacles to facing those challenges?
    c. What does "success" look like in negotiating those struggles?
    d. How does navigating these moments of struggle point you toward developing an identity that fits your goals and expectations for the doctor you aspire to be?
3. Who do others (i.e., the institution, peers, supervisors, family, patients) expect you to become as a doctor? Describe a situation where you felt that the expectations of others did not align with your expectations for who you are striving to become as a doctor. How did this affect you? How did you navigate this conflict? How did you work through your response to those expectations?
4. What does it mean to adopt a new identity as a doctor, particularly as you go through residency training? What happens when this new identity does not fit well right away?
    a. Describe a time where you felt that you did not yet fit into your developing identity as a doctor. What was the situation? What were the challenges? How did you work through those challenges?
    b. What are things like now? What has changed?
5. In what ways does your training support you in becoming the good doctor you imagined yourself to be? In what ways does it hamper you in becoming the good doctor you imagined yourself to be? How much control do you feel that you have in choosing what it means to be a good doctor? In choosing how you individually express what it means to be a good doctor? In choosing your pathway to becoming the type of doctor that you want to become?
6. When you reflect on identity struggles that you have faced during residency training:
    a. Can you provide a specific example of a time when you think you were successful at navigating an identity struggle? How did that affect you as a person? How did that affect you as a doctor?
    b. Can you provide a specific example of a time when you think you were unsuccessful at negotiating that tension? How did that affect you as a person? How did that affect you as a doctor?
